# Association of Anthropometric Indices of Obesity with Hypertension in Chinese Elderly: An Analysis of Age and Gender Differences

**DOI:** 10.3390/ijerph15040801

**Published:** 2018-04-19

**Authors:** Qian Wang, Lingzhong Xu, Jiajia Li, Long Sun, Wenzhe Qin, Gan Ding, Jing Zhu, Jiao Zhang, Zihang Yu, Su Xie

**Affiliations:** 1 School of Public Health, Shandong University, Jinan 250012, China; wangqian0519@126.com (Q.W.); lijiajia@sdu.edu.cn (J.L.); sunlong@sdu.edu.cn (L.S.); qinwenzhe09@163.com (W.Q.); dinggan90@163.com (G.D.); zj1176559133@163.com (Jin.Z); 201614324@mail.sdu.edu.cn (Jia.Z.); 13249220350@163.com (Z.Y.); xs340823@163.com (S.X.); 2 Collaborative Innovation Center of Social Risks Governance in Health, School of Public Health, Fudan University, Shanghai 200032, China

**Keywords:** hypertension, anthropometric indices, obesity, elderly, China

## Abstract

This study aims to explore the association of anthropometric indices of obesity with hypertension in Chinese elderly and its possible gender and age differences. A total of 7070 adults age 60 or older were interviewed in a cross-sectional study conducted in 2017. Anthropometric indices for each participant were measured by using standard methods of trained doctoral/master students. We performed two binary logistic regression models to examine the association of the nine different anthropometric indices and hypertension by gender. Lastly, analyses were performed in two steps stratified for age. Comparing individuals with and without hypertension, there were statistically significant differences in anthropometric indices except height, a body shape index (ABSI), and hip index (HI) in males; and except height in females. There were gender differences in the relationship between anthropometric indices and the prevalence of hypertension in Chinese older adults. After stratification by age, the associations of all anthropometric indices became weaker, disappeared, or even went in the opposite direction. Furthermore, body mass index (BMI) in men (except individuals older than 80) and hip circumference (HC) in women showed a significant impact on the risk of hypertension. The association of anthropometric indices of obesity with hypertension in Chinese elderly differ by gender and age. These findings indicate a need to develop gender-specific strategies for the male and female elderly in the primary and secondary prevention of hypertension.

## 1. Introduction

Hypertension is the leading global risk factor of cardiovascular disease (CVD). It contributes to nearly 9.4 million deaths worldwide annually [1,2]. In China, hypertension is common and its prevalence is rising. Every year, almost 2.1 million cardiovascular deaths and 1.2 million premature cardiovascular deaths are attributed to hypertension [3]. A recent study showed that nearly half of Chinese adults ages 35–75 have hypertension. Among these hypertensive patients, the elderly—aged 60 or older—accounted for 50.1% higher than the sum of other age groups [4].

Obesity is increasing globally and has received widespread attention [5]. Data from the World Health Organization estimated that more than 1.9 billion adults were overweight in 2016, and that more than 650 million were obese [6]. Many studies have suggested that the high incidence of hypertension is associated with the dramatic increase in the prevalence of obesity [7,8,9,10]. Other studies have identified several mechanisms as potential causes of obesity-hypertension to verify this association [11,12,13].

Obesity can be defined by different anthropometric measurements and indices. In recent years, there has been increasing speculation over which measure of obesity predicts CVD better [14]. However, previous studies on this remain controversial. Body mass index (BMI) is commonly used in many epidemiologic studies on obesity. However, it is reported to be incapable of differentiating body fat and lean body mass in many studies [15,16,17,18]. Some studies found statistical evidence that supports the superiority of measures of centralized obesity (such as waist circumference (WC), waist-to-hip ratio (WHR), or waist-to-height ratio (WHtR)) over BMI, for detecting cardiovascular risk factors in both men and women [14,19,20,21,22,23]. Others found that BMI and central obesity indicators were equally well regardless of age and sex [24]. Moreover, many new anthropometric indices were developed recently, such as a new hip index (HI) and a body shape index (ABSI), and these measures proved to be complementary to BMI and other known risk factors [25,26]. Therefore, in our current study, we included more indices (such as height, weight, WC, hip circumference (HC), WHR, WHtR, BMI, HI, and ABSI) than previous studies to make our results more comprehensive.

Aging population has become a worldwide phenomenon. According to China National Bureau of Statistics, the population age 60 and above accounted for 16.2% of the total population at the end of 2015 [27]. Furthermore, hypertension mostly affects elderly people, these individuals are more likely to have organ damages or clinical CVD [4]. However, the majority of studies on the association between anthropometric indices and hypertension to date have targeted general adults rather than the elderly [15,16,17,18,20,21,22,23,24]. Thus, studies focused on these vulnerable population are more necessary.

Although there have been some studies aimed to explore which anthropometric indicators are more strongly associated with hypertension, controversies exist within the results. For example, some studies reported that general obesity (BMI) rather than abdominal obesity linked with hypertension directly [15]; some studies found the superiority of abdominal obesity (such as WC, WHtR) in detecting hypertension [14,19]; and others indicated that these indicators were equally strongly associated with hypertension [24]. In China, many studies explored the association between anthropometric indicators and hypertension from a different gender perspective and tried to find out the better predictor for hypertension in males and females [28,29]. However, few studies stratified for age at the same time, which may significantly influence the association and its gender differences. Therefore, in this study we aim to: (1) explore the association of anthropometric indices of obesity with hypertension among Chinese elderly; (2) find out its possible gender and age differences.

## 2. Materials and Methods

### 2.1. Study Participants and Data Collection

**Figure 1:**
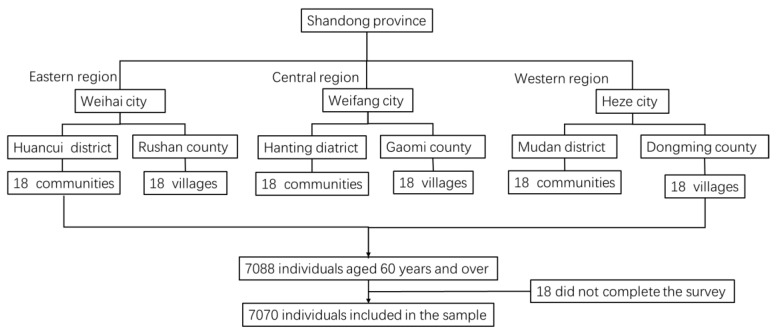
Flow chart of participant enrolment.

This study was collected from the 2017 Survey of the Shandong Elderly Family Health Service, which was conducted by Shandong University. Stratified multi-stage random sampling was applied: in the first stage, six counties were selected from 137 counties as the primary sampling units (PSUs) throughout the eastern, central and western regions of Shandong province (which were divided into three districts and three counties that represented urban and rural areas separately). From each PSU, 18 villages in rural area and 18 communities in suburban and urban area were selected as the secondary sampling units (SSUs). In the third stage, based on the roster of the residents by age and total elderly population of each selected site provided by the local residential committee, an average of 66 individuals were stratified and randomly selected from each SSU making up the total sample. The eligible participants for this survey were those age 60 or older with local household registrations at the time of the interview. Initially, 7088 elderly individuals were selected and interviewed. Of these, 18 did not complete the survey. Finally, a total of 7070 individuals were included in the sample (Figure 1).

All data were collected in the participant’s home using a study questionnaire by trained doctoral/master students. Informed consent for the collection and use of information was obtained from all participants. Medical ethics approval was not required since particular treatments or interventions were not offered or withheld from respondents as a consequence of participating in the study.

### 2.2. Dependent Variable

The elderly with hypertension were self-reported, which derived from a question “Have you been diagnosed with hypertension by a doctor”. If they said “yes,” our investigators (master/doctor students with medical background) checked further. The inclusion criteria were: (i) have been diagnosed with hypertension by doctor; (ii) need to be on long-term medication. Temporary increases of blood pressure were excluded carefully.

### 2.3. Anthropometric Measurements

To obtain a more reliable result, our study included most of the traditional anthropometric indices (height, weight, WC, HC, WHR, WHtR, BMI) and some newly developed anthropometric indices (HI and ABSI).

Height without shoes was measured in centimeters (accuracy 1.0 cm), and weight in light clothing was measured in kilograms (accuracy 0.01 kg). Height was classified into quartiles: Quartile 1 (<154), Quartile 2 (154–160), Quartile 3 (160–165), and Quartile 4 (≥165). Weight was also classified into quartiles: Quartile 1 (<55), Quartile 2 (55–62), Quartile 3 (62–70), and Quartile 4 (≥70).

WC was measured at the level midway between the lower rib margin and the iliac crest, with participants in standing position without heavy outer garments and with emptied out pockets, breathing out gently (accuracy 1 cm). The cut-off points for abdominal obesity were chosen as a WC ≥90 cm for men and ≥80 cm for women [30].

HC was recorded as the maximum circumference over the buttocks (accuracy 1 cm). HC was classified into quartiles: Quartile 1 (<92), Quartile 2 (92–98), Quartile 3 (98–103), and Quartile 4 (≥103).

BMI was calculated as weight divided by height squared (kg/m^2^). BMI was classified based on WHO guidelines as follows: underweight was a BMI < 18.50; normal weight was a BMI of 18.5–24.9; overweight was a BMI of 25.0–29.9; obese was a BMI ≥ 30.0 [31].

WHR was calculated as WC divided by HC. The cut-off value for central obesity was considered ≥0.8 for females and ≥0.9 for males [29].

WHtR was calculated as WC divided by height. The cut-off value for central obesity was considered ≥0.5 for both males and females [32].

ABSI based on WC adjusted for height and weight was developed by Krakauer N.Y. et al. [26].

$$\mathrm{ABSI}=\frac{\mathrm{WC}}{{\mathrm{BMI}}^{\frac{2}{3}}{\mathrm{height}}^{\frac{1}{2}}}$$

HI based on HC, height and weight was developed by Krakauer N.Y. et al. [25]

$$\mathrm{HI}=\mathrm{HC}{\left(\frac{H}{\langle H\rangle }\right)}^{0.310}{\left(\frac{W}{\langle W\rangle }\right)}^{-0.428}$$

where 〈*H*〉 = 166 cm and 〈*W*〉 = 73 kg.

ABSI and HI were both classified into quartiles: Quartile 1 was the lowest value and Quartile 4 (Q4) was the highest value.

### 2.4. Covariates

Socio-demographic factors included gender (male, female), age (60–64, 65–69, 70–74, 75–79, ≥80), marital status (single, married), empty nest (empty nest, non-empty nest), education level (no formal education, primary education, secondary or above), employment (employed, unemployed), and personal income (0–2500, 2500–5000, 5000–15,000, ≥15,000 RMB Yuan). Health behavior-related factors included smoking status (nonsmoker, ex-smoker, current smoker) and drinking status (never drink, past drink, drink seldom, drink occasionally, drink more often). Health-related factors included self-rated health (very bad, bad, moderate, very good, excellent) and activities of daily living (ADL). Activities of daily living were assessed by using the Activities of Daily Living Scale (ADLs), which includes physical self-maintenance scale (PSMS) and instrumental activities of daily living scale (IADL). The ADL scale is used to evaluate people’s simple and basic ability to practice one’s normal life independently. The reliability and validity of ADL instrument in Chinese-language version was verified to be good [33]. Scores of ADL can be divided into three levels: no obvious dysfunction (≤14 scores), functional decline (15–21 scores), and obvious dysfunction (≥22 scores).

### 2.5. Statistical Analysis

We used SPSS 24.0 (IBM Corp, Armonk, NY, USA) to analyze the data. The socio-demographic characteristics of individuals with and without hypertension were compared by chi-square for categorical variables. Distributions of anthropometric indices between hypertensive and normotensive participants in both males and females were compared by chi-square for categorical variables. Binary logistic regression models with an enter method were used to examine the independent association of the nine different anthropometric indices and hypertension by gender. Model 1: binary logistic regression model by gender, a basic model adjusted for socio-demographic factors characteristics (gender, age, marital status, education level, employment, personal income, smoking status, drinking status, empty nest) and health-related factors (self-rated health, ADL scores), entered each time one of the anthropometric indices that show significant difference in chi-square. Model 2: binary logistic regression model by gender, the basic model further adjusted for all the other anthropometric indices that show significant difference in model 1. Associations were determined by odds ratios (ORs) and 95% confidence intervals. In addition, logistic regression analyses were also performed in two steps separately for male and female subjects and stratified for each 10-year age group. In the first step, each of the anthropometric indices was included as independent variables, adjusted for all the covariates. In the second step, the statistical anthropometric indices in step one included as independent variables, also adjusted for all the covariates. The statistical significance in this paper was set at the 5% level.

## 3. Results

Table 1 shows the demographic data of the study subjects. The study included 7070 respondents with a mean age of 69.81 (±6.445) years. The prevalence of hypertension was 44.19%, 39.0% in males and 47.7% in females. Generally, the majority of the participants had the following characteristics: female (59.7%), married (81.2%), a primary or below education level (73.5%), unemployed (69.2%), nonsmokers (71.1%), nondrinkers (75.9%), and non-empty nest (72.8%). As for health characteristics, most of the elderly reported moderate and above health (81.7%) and with no obvious dysfunction (77.3%).

Distributions of anthropometric variables in males and females were showed as follows. Comparing individuals with and without hypertension, there were statistically significant differences in anthropometric indices except height, ABSI, and HI in males, and except height in females (Table 2 and Table 3).

The results of binary logistic regression by gender are shown in Table 4. The results of model 1 showed that the association of weight, WC, HC, WHR, WHtR, and BMI with hypertension were statistically significant in both males and females, and that two new developed indicators (ABSI and HI) were statistically significant in females. After adjusting for other anthropometric indices that have statistical significance in model 1, WC and BMI were still statistically significant in the association with hypertension in males, HC, BMI, and HI were still statistically significant in females. Compared with the reference group, individuals with a larger WC or a higher BMI were associated with a greater risk for having hypertension in male, and those with a larger HC, a higher BMI or a larger HI were associated with a greater risk for having hypertension in females.

The logistic regression analyses stratified for age were conducted in two steps. After the first step (data not shown), anthropometric indices that had statistical significance were chosen from each age group in both males and females, and entered in the second step (Table 5). Adjustment for these anthropometric indices and all the covariates affected all associations. Among the male elderly, only WC and BMI were statistical significant in the association with hypertension in the 60–70 year old group, and only BMI was statistically significant in the 70–80 year old group. However, there were no statistical significance in the relationship between hypertension and anthropometric indices in the male elderly older than 80. Among the female elderly, only HC, WHtR, and BMI were statistically significant in the association with hypertension in the 60–70 year old group; only HC, HI, ABSI, and BMI were statistically significant in the 70–80 year old group; and only HC was statistically significant in the older than 80 year old group. In these older age groups, the associations of all anthropometric indices and hypertension became weaker, disappeared or even went in the opposite direction. 

## 4. Discussion

Our research showed that there were significant differences in most of the anthropometric indices between hypertensive patients and normotensive individuals, including both overall and abdominal obesity indicators. These results suggested that hypertension is linked to obesity in both male and female elderly, which is inconsistent with previous studies that both overall and abdominal obesity are significantly associated with hypertension [34,35]. The mechanisms of this association include structural arterial abnormalities, leptin, and the activation of the renin–angiotensin–aldosterone axis [13,36]. Furthermore, other studies showed that it may also be related to the variant of gene [37,38]. Fortunately, obesity being a modifiable factor, lifestyle modifications including increased physical activity, endurance and endurance-strength exercise, and dietary modifications can decrease the incidence of hypertension [39,40,41].

In the present study, we found that there were gender differences and age differences in the relationship between anthropometric indices and the prevalence of hypertension in Chinese elderly. In both genders, association was stronger between BMI and hypertension without considering the impact of age. These results are supported by numerous studies that the increase of BMI contributes to blood pressure increase [42,43,44]. After we took age into account, the association of BMI and hypertension in both males and females was weaker in the older elderly compared to those younger ones. However, the association of HI and hypertension in females was stronger with increased age.

After stratified by gender and age, BMI in men (except individuals older than 80) and HC in women remained significant in the relationship with hypertension in all age groups. Our findings in males are consistent with previous studies that BMI have the highest correlation with hypertension [45,46]. Besides, analyses of our findings revealed a positive relationship between HC and the incidence of hypertension in females. This relationship is inconsistent with most of the previous studies [46,47]. However, there are still some studies support us that high HC is a risk factor for the multi-metabolic disorders [48,49,50].This result may be due to the following two reasons. First, HC carries some information on both overall obesity and abdominal obesity, since HC is positively correlated with BMI and WC [48]. Second, older Chinese women with a higher HC usually have experienced gravidity more times, studies have indicated that more gravidity was associated with a consistent increase in the risk of metabolic syndrome in older Chinese women [51,52]. In view of these results, it should be a priority to encourage those males with high BMI and females with high HC to pay more attention to guidelines of hypertension. Besides, health care facilities should strengthen the screening and monitoring for those at-risk subgroups to early identify and treat the elderly with hypertension.

The main results of the current study indicated that, overall, obesity was associated with hypertension in males, and partial obesity was associated with hypertension in females. This may be concluded that the amount of body fat mass in males and the distribution of body fat in females is closely related to hypertension [16]. In previous studies, general obesity and partial obesity usually showed the similar relationship with hypertension in different gender. For example, some studies indicated that general obesity has a stronger association with hypertension in both males and females [15,24]. Others found that the association of partial obesity with hypertension was stronger in both genders [14,19]. Our results indicated that the role of overall obesity and partial obesity in hypertension in separate gender may be different. Further studies are necessary to confirm this assumption.

This study has a number of strengths. First, we included more anthropometric indices than previous studies, which made our results more comprehensive. Second, to our best knowledge, this is the first study to analyze the gender and age difference of association between anthropometric indices and hypertension in Chinese elderly. Furthermore, the sample size in this study was relatively large, which makes our results more reliable.

However, there are some limitations that should be considered while interpreting the results. First, the cross-sectional design does not allow for causal inferences to be made about the relationships of obesity with the prevalence of hypertension. However, the largely causal association between obesity and hypertension is well established [53]. Thus, it can be speculated that excess obesity precedes hypertension. Second, there may be more confounding factors than those available for consideration in our study. Third, using cut points for defining the metabolic disorders implies a loss of information, and more studies are needed in order to find out the relationships between anthropometric indices and continuous metabolic variables by use of linear regression.

## 5. Conclusions

In summary, there are age and gender differences in the association between anthropometric indices of obesity and hypertension in Chinese elderly. Moreover, BMI in males (except individuals aged ≥ 80 years) and HC in females were found to have a significant association with hypertension in all age groups.

These findings indicate a need to develop gender-specific strategies for the male and female elderly in the primary and secondary prevention of hypertension.

## Figures and Tables

**Table 1:** Characteristics between hypertensive and normotensive participants.

Characteristics	Total	Hypertensive	Normotensive	X^2^	*p*
	(*n* = 7070) *n* (%)	(*n* = 3124) *n* (%)	(*n* = 3946) *n* (%)		
Gender				51.920	0.000
Male	2846 (40.3)	1110 (39.0)	1736 (61.0)		
Female	4224 (59.7)	2014 (47.7)	2210 (52.3)		
Age				83.129	0.000
60–64	1577 (22.3)	561 (35.6)	1016 (64.4)		
65–69	2129 (30.1)	941 (44.2)	1188 (55.8)		
70–74	1780 (25.2)	898 (50.4)	882 (49.6)		
75–80	975 (13.8)	469 (48.1)	506 (51.9)		
≥80	609 (8.6)	255 (41.9)	354 (58.1)		
Residence				0.385	0.825
Rural	4990 (70.6)	2203 (44.1)	2787 (55.9)		
Township	524 (7.4)	226 (43.1)	298 (56.9)		
City	1556 (22.0)	695 (44.7)	861 (55.3)		
Marital status				19.931	0.000
Single	1331 (18.8)	661 (49.7)	670 (50.3)		
Married	5739 (81.2)	2463 (42.9)	3276 (57.1)		
Education level				18.985	0.000
No formal education	2270 (32.1)	1083 (47.7)	1187 (52.3)		
Primary education	2924 (41.4)	1268 (43.4)	1656 (56.6)		
Secondary or above	1876 (26.5)	773 (41.2)	1103 (58.8)		
Employment				21.085	0.000
Employed	2176 (30.8)	873 (40.1)	1303 (59.9)		
Unemployed	4894 (69.2)	2251 (46.0)	2643 (54.0)		
Personal income				10.436	0.015
0–2500	1571 (22.2)	673 (42.8)	898 (57.2)		
2500–5000	1689 (23.9)	792 (46.9)	897 (53.1)		
5000–15,000	1908 (27.0)	860 (45.1)	1048 (54.9)		
≥15,000	1902 (26.9)	799 (42.0)	1103 (58.0)		
Smoking status				72.709	0.000
Nonsmoker	5029 (71.1)	2334 (46.4)	2695 (53.6)		
Ex-smoker	903 (12.8)	418 (46.3)	485 (53.7)		
Current smoker	1138 (16.1)	372 (32.7)	766 (67.3)		
Drinking status				57.511	0.000
Never drink	5365 (75.9)	2453 (45.7)	2912 (54.3)		
Past drink	552 (7.8)	275 (49.8)	277 (50.2)		
Drink seldom	377 (16.3)	126 (33.4)	251 (66.6)		
Drink occasionally	98 (1.4)	33 (33.7)	65 (66.3)		
Drink more often	678 (9.6)	237 (35.0)	441 (65.0)		
Exercise time (per day)				3.779	0.286
Never	2719 (38.5)	1182 (43.5)	1537 (56.5)		
Less than half hour	2160 (30.6)	966 (44.7)	1194 (55.3)		
More than half hour	1547 (21.9)	707 (45.7)	840 (54.3)		
More than one hour	644 (9.1)	269 (41.8)	375 (58.2)		
Empty nest				5.790	0.016
Empty nest	1923 (27.2)	805 (41.9)	1118 (58.1)		
Non-empty nest	5147 (72.8)	2319 (45.1)	2828 (54.9)		
Self-rated health				336.988	0.000
Very bad	127 (1.8)	74 (58.3)	53 (41.7)		
Bad	1169 (16.5)	667 (57.1)	502 (42.9)		
Moderate	1992 (28.2)	1051 (52.8)	941 (47.2)		
Very good	2639 (37.3)	1043 (39.5)	1596 (60.5)		
Excellent	1143 (16.2)	289 (25.3)	854 (74.7)		
ADL scores				57.648	0.000
14	5467 (77.3)	2291 (41.9)	3176 (58.1)		
15–21	1283 (18.1)	646 (50.4)	637 (49.6)		
22+	320 (4.5)	187 (58.4)	133 (41.6)		

**Table 2:** Distributions of anthropometric variables in males

Characteristics	Total	Hypertension	Normotensive	X^2^	*p*
	(*n* = 2846) *n* (%)	(*n* = 1110) *n* (%)	(*n* = 1736) *n* (%)		
Height				0.256	0.968
Q1	162 (5.7)	61 (37.7)	101 (62.3)		
Q2	554 (19.5)	213 (38.4)	341 (61.6)		
Q3	701 (24.6)	276 (39.4)	425 (60.6)		
Q4	1429 (50.2)	560 (39.2)	869 (60.8)		
Weight				89.441	0.000
Q1	490 (17.2)	128 (26.1)	362 (73.9)		
Q2	630 (22.1)	212 (33.7)	418 (66.3)		
Q3	891 (31.3)	347 (38.9)	544 (61.1)		
Q4	835 (29.3)	423 (50.7)	412 (49.3)		
WC				106.522	0.000
<90	831 (29.2)	202 (24.3)	629 (75.7)		
≥90	2015 (70.8)	908 (45.1)	1107 (54.9)		
HC				91.743	0.000
Q1	701 (24.6)	187 (26.7)	514 (73.3)		
Q2	774 (27.2)	280 (36.2)	494 (63.8)		
Q3	640 (22.5)	274 (42.8)	366 (57.2)		
Q4	731 (25.7)	369 (50.5)	362 (49.5)		
WHR				27.46	0.000
<0.9	713 (25.1)	219 (30.7)	494 (69.3)		
≥0.9	2133 (74.9)	891 (41.8)	1242 (58.2)		
WHtR				81.806	0.000
<0.5	612 (21.5)	142 (23.2)	470 (76.8)		
≥0.5	2234 (78.5)	968 (43.3)	1266 (56.7)		
BMI				107.422	0.000
Underweight	122 (4.3)	23 (18.9)	99 (81.1)		
Normal weight	1684 (59.2)	568 (33.7)	1116 (66.3)		
Overweight	910 (32.0)	434 (47.7)	476 (52.3)		
Obese	130 (4.6)	85 (65.4)	45 (34.6)		
ABSI				5.513	0.138
Q1	693 (24.3)	253 (36.5)	440 (63.5)		
Q2	786 (27.6)	296 (37.7)	490 (62.3)		
Q3	735 (25.8)	294 (40.0)	441 (60.0)		
Q4	632 (22.2)	267 (42.2)	365 (57.8)		
HI				3.228	0.358
Q1	974 (34.2)	363 (37.3)	611 (62.7)		
Q2	913 (32.1)	353 (38.7)	560 (61.3)		
Q3	585 (20.6)	237 (40.5)	348 (59.5)		
Q4	374 (13.1)	157 (42.0)	217 (58.0)		

Note: WC = waist circumference (cm), HC = hip circumference (cm), WHR = waist to hip ratio, WHtR = waist to height ratio, BMI = body mass index (kg/m^2^), ABSI = a body shape index, HI = hip index.

**Table 3:** Distributions of anthropometric variables in females

Characteristics	Total	Hypertension	Normotensive	X^2^	*p*
	(*n* = 4224) *n* (%)	(*n* = 2014) *n* (%)	(*n* = 2210) *n* (%)		
Height				2.326	0.508
Q1	1713 (40.6)	834 (48.7)	879 (51.3)		
Q2	1807 (42.8)	860 (47.6)	947 (52.4)		
Q3	560 (13.3)	252 (45.0)	308 (55.0)		
Q4	144 (3.4)	68 (47.2)	76 (52.8)		
Weight				79.705	0.000
Q1	1324 (31.3)	516 (39.0)	808 (61.0)		
Q2	1129 (26.7)	523 (46.3)	606 (53.7)		
Q3	1110 (26.3)	613 (55.2)	497 (44.8)		
Q4	661 (15.6)	362 (54.8)	299 (45.2)		
WC				83.932	0.000
<80	527 (12.5)	153 (29.0)	374 (71.0)		
≥80	3697 (87.5)	1861 (50.3)	1836 (49.7)		
HC				107.647	0.000
Q1	929 (22.0)	324 (34.9)	605 (65.1)		
Q2	1042 (24.7)	469 (45.0)	573 (55.0)		
Q3	939 (22.2)	482 (51.3)	457 (48.7)		
Q4	1314 (31.1)	739 (56.2)	575 (43.8)		
WHR				8.319	0.004
<0.8	74 (1.8)	23 (31.1)	51 (68.9)		
≥0.8	4150 (98.2)	1991 (48.0)	2159 (52.0)		
WHtR				77.75	0.000
<0.5	395 (9.4)	105 (26.6)	290 (73.4)		
≥0.5	3829 (90.6)	1909 (49.9)	1920 (50.1)		
BMI				112.593	0.000
Underweight	112 (2.7)	25 (22.3)	87 (77.7)		
Normal weight	2001 (47.4)	832 (41.6)	1169 (58.4)		
Overweight	1716 (40.6)	911 (53.1)	805 (46.9)		
Obese	395 (9.4)	246 (62.3)	149 (37.7)		
ABSI				14.533	0.002
Q1	1080 (25.6)	469 (43.4)	611 (56.6)		
Q2	1006 (23.8)	481 (47.8)	525 (52.2)		
Q3	991 (23.5)	513 (51.8)	478 (48.2)		
Q4	1147 (27.2)	551 (48.0)	596 (52.0)		
HI				10.582	0.014
Q1	792 (18.8)	348 (43.9)	444 (56.1)		
Q2	857 (20.3)	410 (47.8)	447 (52.2)		
Q3	1179 (27.9)	603 (51.1)	576 (48.9)		
Q4	1396 (33.0)	653 (46.8)	743 (53.2)		

Note: WC = waist circumference (cm), HC = hip circumference (cm), WHR = waist to hip ratio, WHtR = waist to height ratio, BMI = body mass index (kg/m^2^), ABSI = A body shape index, HI = hip index.

**Table 4:** The relationship between hypertension and anthropometric variables in both males and females

Characteristics	Model 1	*p*	Female (*n* = 4224)	*p*	Model 2	*p*	Female (*n* = 4224)	*p*
	Male (*n* = 2846)				Male (*n* = 2846)			
Weight ^a^		**0.000**		**0.000**		0.224		0.205
Q2	**1.609 (1.223, 2.115)**	**0.001**	**1.502 (1.268, 1.779)**	**0.000**	1.120 (0.817, 1.534)	0.481	0.964 (0.778, 1.195)	0.738
Q3	**2.125 (1.642, 2.750)**	**0.000**	**2.241 (1.884, 2.667)**	**0.000**	1.117 (0.792, 1.575)	0.527	1.083 (0.810, 1.449)	0.589
Q4	**3.590 (2.749, 4.688)**	**0.000**	**2.283 (1.861, 2.800)**	**0.000**	1.436 (0.949, 2.174)	0.087	0.850 (0.586, 1.232)	0.390
WC	**2.716 (2.240, 3.293)**	**0.000**	**2.497 (2.034, 3.066)**	**0.000**	**1.936 (1.360, 2.756)**	**0.000**	1.257 (0.899, 1.757)	0.182
HC ^a^		**0.000**		**0.000**		0.898		**0.003**
Q2	**1.620 (1.283, 2.044)**	**0.000**	**1.600 (1.325, 1.933)**	**0.000**	0.922 (0.679, 1.252)	0.605	**1.356 (1.076, 1.708)**	**0.010**
Q3	**2.143 (1.681, 2.732)**	**0.000**	**2.103 (1.731, 2.555)**	**0.000**	0.889 (0.618, 1.277)	0.524	**1.682 (1.255, 2.253)**	**0.000**
Q4	**2.923 (2.305, 3.708)**	**0.000**	**2.584 (2.153, 3.101)**	**0.000**	0.947 (0.642, 1.396)	0.782	**1.972 (1.371, 2.836)**	**0.000**
WHR	**1.623 (1.343, 1.961)**	**0.000**	**1.863 (1.116, 3.110)**	**0.017**	0.976 (0.774, 1.230)	0.837	0.884 (0.500, 1.565)	0.673
WHtR	**2.509 (2.022, 3.113)**	**0.000**	**2.654 (2.090, 3.371)**	**0.000**	1.166 (0.834, 1.631)	0.368	1.311 (0.905, 1.899)	0.152
BMI ^b^		**0.000**		**0.000**		**0.000**		**0.004**
Normal weight	**2.599 (1.600, 4.223)**	**0.000**	**2.888 (1.816, 4.593)**	**0.000**	1.693 (0.991, 2.890)	0.054	**1.713 (1.037, 2.828)**	**0.035**
Overweight	**4.617 (2.811, 7.583)**	**0.000**	**4.898 (3.068, 7.818)**	**0.000**	**2.026 (1.124, 3.652)**	**0.019**	**2.055 (1.188, 3.555)**	**0.010**
Obese	**10.682 (5.813, 19.631)**	**0.000**	**6.819 (4.123, 11.280)**	**0.000**	**4.327 (2.138, 8.758)**	**0.000**	**2.927 (1.584, 5.410)**	**0.001**
ABSI ^c^				**0.036**				0.388
Q2			1.136 (0.950, 1.360)	0.163			1.024 (0.841, 1.247)	0.813
Q3			**1.292 (1.078, 1.549)**	**0.006**			1.162 (0.942, 1.434)	0.161
Q4			1.063 (0.889, 1.271)	0.503			1.018 (0.813, 1.276)	0.875
HI ^c^				**0.031**				**0.028**
Q2			1.170 (0.956, 1.432)	0.128			0.902 (0.714, 1.138)	0.384
Q3			**1.281 (1.061, 1.547)**	**0.010**			0.910 (0.707, 1.172)	0.464
Q4			1.052 (0.875, 1.263)	0.590			**0.701 (0.521, 0.943)**	**0.019**

Note: The bold indicate significance. WC = waist circumference (cm), HC = hip circumference (cm), WHR = waist to hip ratio, WHtR = waist to height ratio, BMI = body mass index (kg/m^2^), ABSI = A body shape index, HI = hip index; ^a^ Reference category is Q1. ^b^ Reference category is underweight. ^c^ Reference category is Q4.

**Table 5:** The relationship between hypertension and anthropometric variables in both males and females stratified by age

Characteristics	Male	70–80 (*n* = 1199)	80+ (*n* = 261)	Female	70–80 (*n* = 1556)	80+ (*n* = 348)
	60–70 (*n* = 1386)			60–70 (*n* = 2320)		
Weight ^a^						
Q2	1.076 (0.634–1.827)	1.446 (0.918–2.276)	0.427 (0.145–1.255)	1.042 (0.786–1.383)	0.952 (0.672–1.349)	1.309 (0.663–2.581)
Q3	1.099 (0.621–1.944)	1.346 (0.816–2.220)	0.898 (0.261–3.083)	1.194 (0.829–1.718)	1.113 (0.689–1.799)	0.894 (0.335–2.389)
Q4	1.501 (0.774–2.911)	1.639 (0.885–3.036)	1.095 (0.230–5.217)	0.896 (0.575–1.394)	1.000 (0.533–1.876)	2.650 (0.324–21.683)
WC	2.454 (1.423–4.233) **	1.454 (0.862–2.452)	2.578 (0.802–8.284)	0.927 (0.576–1.492)	1.718 (0.980–3.011)	1.286 (0.504–3.280)
HC ^a^						
Q2	0.873 (0.545–1.397)	0.821 (0.521–1.292)	2.094 (0.652–6.726)	1.263 (0.935–1.707)	1.507 (1.028–2.209) **	1.009 (0.532–1.912)
Q3	0.954 (0.554–1.644)	0.730 (0.422–1.265)	0.989 (0.226–4.323)	1.389 (1.001–1.928) **	1.992 (1.228–3.231) **	1.199 (0.517–2.781)
Q4	0.794 (0.442–1.429)	0.989 (0.549–1.781)	1.257 (0.237–6.676)	1.331 (0.935–1.896)	2.364 (1.287–4.342) **	2.655 (1.142–6.169) **
WHR	0.791 (0.562–1.114)	1.218 (0.854–1.738)		1.023 (0.491–2.131)		
WHtR	1.059 (0.637–1.761)	1.228 (0.748–2.018)	1.474 (0.424–5.126)	1.727 (1.033–2.887) **	0.766 (0.406–1.445)	1.457 (0.514–4.134)
BMI ^b^						
Normal weight	2.032 (0.735–5.616)	1.665 (0.813–3.410)		1.628 (0.725–3.654)	2.181 (0.988–4.815)	1.172 (0.390–3.522)
Overweight	2.588 (0.876–7.644)	1.934 (0.862–4.341)		2.602 (1.103–6.139) **	2.077 (0.871–4.948)	0.855 (0.229–3.197)
Obese	6.745 (1.975–23.040) **	4.372 (1.582–12.082) **		3.614 (1.441–9.067) **	3.049 (1.123–8.281) **	3.563 (0.488–26.005)
ABSI ^c^						
Q2					1.191 (0.841–1.686)	
Q3					1.466 (1.025–2.097) **	
Q4					1.179 (0.808–1.720)	
HI ^c^						
Q2			1.233 (0.491–3.097)		0.792 (0.538–1.166)	
Q3			1.123 (0.406–3.108)		0.869 (0.572–1.321)	
Q4			2.322 (0.653–8.262)		0.553 (0.341–0.898) **	

Note: The bold indicate significance. WC = waist circumference (cm), HC = hip circumference (cm), WHR = waist to hip ratio, WHtR = waist to height ratio, BMI = body mass index (kg/m^2^), ABSI = A body shape index, HI = hip index. ^a^ Reference category is Q1. ^b^ Reference category is underweight. ^c^ Reference category is Q4. **p < 0.05.

## Abbreviations

BMI	body mass index
HC	hip circumference
CVD	cardiovascular disease
WC	waist circumference
WHR	waist-to-hip ratio
WHtR	waist-to-height
HI	hip index
ABSI	body shape index
PSUs	primary sampling units
SSUs	secondary sampling units
ADL	activities of daily living
ADLs	activities of daily living scale
IADL	instrumental activities of daily living scale
PSMS	physical self-maintenance scale
IADL	activities of daily living scale
ORs	odds ratios
